# A Monetary Reward Alters Pacing but Not Performance in Competitive Cyclists

**DOI:** 10.3389/fphys.2017.00741

**Published:** 2017-09-29

**Authors:** Sabrina Skorski, Kevin G. Thompson, Richard J. Keegan, Tim Meyer, Chris R. Abbiss

**Affiliations:** ^1^Institute of Sports and Preventive Medicine, Saarland University, Saarbrücken, Germany; ^2^Research Institute for Sport and Exercise, University of Canberra, Bruce, ACT, Australia; ^3^Centre for Exercise and Sports Science Research, School of Exercise and Health Sciences, Edith Cowan University, Joondalup, WA, Australia

**Keywords:** cycling, time trial, motivation, extrinsic, monetary reward, pacing strategy

## Abstract

Money has frequently been used as an extrinsic motivator since it is assumed that humans are willing to invest more effort for financial reward. However, the influence of a monetary reward on pacing and performance in trained athletes is not well-understood. Therefore, the aim of this study was to analyse the influence of a monetary reward in well-trained cyclists on their pacing and performance during short and long cycling time trials (TT). Twentythree cyclists (6 ♀, 17 ♂) completed 4 self-paced time trials (TTs, 2 short: 4 km and 6 min; 2 long: 20 km and 30 min); in a randomized order. Participants were separated into parallel, non-randomized “rewarded” and “non-rewarded” groups. Cyclists in the rewarded group received a monetary reward based on highest mean power output across all TTs. Cyclists in the non-rewarded group did not receive a monetary reward. Overall performance was not significantly different between groups in short or long TTs (*p* > 0.48). Power output showed moderatly lower effect sizes at comencement of the short TTs (*P*_meandiff_ = 36.6 W; *d* > 0.44) and the 20 km TT (*P*_meandiff_ = 22.6 W; *d* = 0.44) in the rewarded group. No difference was observed in pacing during the 30 min TT (*p* = 0.95). An external reward seems to have influenced pacing at the commencement of time trials. Participants in the non-rewarded group adopted a typical parabolic shaped pattern, whereas participants in the rewarded group started trials more conservatively. Results raise the possibility that using money as an extrinsic reward may interfere with regulatory processes required for effective pacing.

## Introduction

In order to finish a race in the fastest possible time, athletes need to appropriately distribute their energy expenditure in a way that all available energetic resources are used but not so early as to experience premature fatigue prior to the finish line (St. Clair Gibson et al., 2006; Skorski and Abbiss, 2017). In sport science literature this has been termed as “pacing” or “pacing pattern” (Abbiss and Laursen, 2008). Since the differences in “classical” physiological characteristics (e.g., VO_2max_, lactate thresholds) of top-level athletes are diminishing, optimal pacing is becoming increasingly important in research and practice (Abbiss and Laursen, 2008; Hettinga et al., 2017; Skorski and Abbiss, 2017).

Marcora (2010) and Pageaux (2014) recently proposed the Psychobiological Model to predict self-paced endurance performance. This Model is based on the “*Motivational Intensity Theory*” published by Brehm and Self (1989) and theorizes that the regulation of speed and/or power output is determined by five cognitive/motivational factors (Pageaux, 2014): “(i) perception of effort, (ii) potential motivation, (iii) knowledge of the distance/time to cover, (iv) knowledge of the distance/time remaining and (v) previous experience (memory of perception of effort during exercise of varying intensity and duration)” (Brehm and Self, 1989). In this concept, “*potential motivation”* “refers to the maximum effort a person is willing to exert to satisfy a motive,” while “*motivation intensity”* is “the amount of effort that people actually expend” (Wright, 2008). According to the authors, *potential motivation* could be influenced by the external environment; for example athletes might show higher motivation during an event with real competitors as when racing against the clock in a laboratory based test (Pageaux, 2014). Indeed, even though pacing is considered as a stable pattern (Mauger et al., 2009; Thomas et al., 2011) it has been observed that during very important competitions some athletes start their race with an ambitious strategy as they are very motivated to compete with medalists, even though based on previous performances they would not be anticipated to finish near a medal (Hulleman et al., 2007). Additionally, the presence of a competitor can improve performance in head-to-head competitions, possibly due to increased extrinsic motivation (Corbett et al., 2012; Williams et al., 2015).

In this regard, motivation is often considered to be a uniquely human trait (Dijksterhuis and Aarts, 2010), that is generally goal directed and central to self-regulation (Seo et al., 2004). One way of studying motivation has been to focus on the socio-cognitive regulation of motivated behavior. Self-determination theory (SDT) (Ryan and Deci, 2000) proposes different forms of motivational regulation, varying in autonomy from intrinsic to extrinsic. As such intrinsic motivation is defined as “the most self-determined form of motivation,” whereby an individual participates in an activity for the satisfaction inherent within the activity itself (Gucciardi, 2010). Extrinsic motivation refers to “engaging in an activity as a means-to-an-end and not for its own sake” (Ryan and Deci, 2000). In an athletic context it has been shown that motivation can influence exercise performance since athletes with a high degree of self-determined motivation seem to perform better and invest more effort in activities than less motivated athletes (Gucciardi, 2010).

The majority of studies have examined motivational influences using untrained participants, however, trained athletes demonstrate different motivational goals (Corbett et al., 2012), which might influence their response to an external motivator. In research, money has frequently been used as a extrinsic motivator since it has been assumed that humans are willing to invest more effort for financial rewards (Zedelius et al., 2014). However, financial rewards are inconsistently associated with better performance in very simplistic tasks and may in fact be detrimental to performance in more demanding tasks (Bijleveld et al., 2011b; Zedelius et al., 2011). The influence of a monetary reward on performance in a homogeneous group of well-trained athletes is not well-understood. To our knowledge only one study has examined the influence of a monetary reward in trained athletes, observing no effects on either pacing or performance (Hulleman et al., 2007). In this study cyclists were spontaneously (when arriving in the laboratory) rewarded with $100 if they outperformed their best 1,500 m cycling performance by more than 1 s (Hulleman et al., 2007). Since no difference was observed, the authors suggested that the monetary reward might have been too small or been presented too close to the start of the trial to affect pacing and performance (Hulleman et al., 2007). Additionally, elite athletes may deal with extrinsic incentives differently to amateur and recreational athletes, because elite athletes may have successfully “internalized” the inherent reward structures of their sport (Keegan et al., 2014).

There is an increasing need to understand the role of rewards and incentives in athletic performance; in particular, when we consider that almost every real competition offers an extrinsic motivator (e.g., prize money, status, recognition, praise), then understanding these processes will be important for future research to ensure a transfer into elite sports. Therefore, the aim of the present study was to examine the influence of a monetary reward in well-trained athletes on pacing pattern and overall performance in short (4 km and 6 min) and long (20 km and 30 min) distance and duration cycling time trials. A recent companion manuscript by Abbiss et al. (2016) observed that despite similar average power output, cyclists seem to start distance-based trials at a higher power output, when compared with the time-based trials. Furthermore, in an athletic setting, athletes are often required to compete in events of a known distance or perform over a given exercise duration. Additionally, training programming in endurance sports, typically involves the prescription of time-based performance tasks, even for sports dominated by distance-based competition. Thus, to minimize possible influences due to more/less experience in time- and/or distance-based trials we decided to include both types. In light of the unknown relationship between motivational regulation and the regulation of pacing, it was hypothesized that the additional incentive of a monetary reward will not influence pacing and overall performance.

## Materials and methods

### Participants

All particpants provided written informed consent in accordance with the appropriate Human Research Ethics Committee. A total of 23 endurance trained cyclists and triathletes (6 ♀, 17 ♂) volunteered to participate in the study. Twelve participants (non-rewarded group) were based in Perth (Western Australia) and 11 (rewarded group) in Canberra (Australian Capital Territory; Table 1). Some data from the non-reward group of the present study has previously published in a manuscript comparing pacing profiles between time and distance based trials (Abbiss et al., 2016). Based on the guidelines to classify subject groups in sport science (De Pauw et al., 2013) participants were categorized into performance level 3 (PPO: 4.2–4.4 W/kg, training: 250–291 km/week; Table 1).

**Table 1:** Anthropometric and performance characteristics of participants in the rewarded (*n* = 11) and the non-rewarded (*n* = 12) goup.

	**Age (y)**	**Height (cm)**	**Weight (kg)**	**Training volume (km/y)**	**P_max_ (W)**	**P_max_ (W/kg)**
No reward	36.4 ± 7.4	174.5 ± 8.9	73.9 ± 9.7	15,600 ± 7,062	391.2 ± 48.2	4.38 ± 0.4
Reward	36.2 ± 5.8	179.9 ± 9.3	76.6 ± 9.3	13,100 ± 5,253	397.8 ± 41.2	4.24 ± 0.4
Effect size (d)				−0.33 ± 0.73	0.13 ± 0.75	−0.07 ± 0.98

*Mean ± Standard deviation; d ± CL*.

In order to determine the influence of monetary reward on pacing pattern and overall performance, participants were separated into two groups, one of which was provided monetary reward (Canberra) while the other was not (Perth). Participants were seperated based on location to avoid effects caused by participants discovering that others were receiving financial reward. Indeed, the local cycling community is relatively small and close knit. Groups were matched based on their peak power output reached during the incremental cycling test, age and training volume per year. Groups did not differ in any of these parameters (Table 1). Prior to the study, the participants in the rewarded group were informed that a monetary reward would be paid to all participants and that the amount was dependent on their highest average power output relative to body weight (W/kg) produced over all trials. Participants were informed that this payment was to encourage maximal performance from them and were not informed that the purpose of this reward was to examine the influence of external motivation. Participants in the non-reward group did not receive any prize money or any other external reward nor were they informed of any money being given to participants in the other group. Prize money was similar to that offered during club/state based cycling events and was paid from 1st to 11th as follows: AUS$ 350, 200, 150, 110, 90, 80, 70, 50, 30, 20, 7. Participants were not informed about the aim of the study until data collection was completed.

### Experimental protocol

All participants attended the laboratory on five separate occasions. During the first visit they performed an incremental cycling test to exhaustion on an electromagnetic cycle ergometer to determine peak power output (Velotron, RacerMate, Seattle, WA). Power output began at 70 W for 1 min, after which power output was progressively increased by 35 W every 1 min until volitional exhaustion. Following this, and in a randomized order, participants performed 4 self-paced cycling time trials of varying duration and distance with 3–7 days between trials. These experimental trials included 2 long (20 km and 30 min) and 2 short (4 km and 6 min) time trials. Participants were asked to refrain from strenuous exercise in the 48 h prior to each trial and then replicate their training load and nutrition (including caffeine) as closely as possible before each trial (controlled by means of written training diaries). Each experimental trial was conducted at approximately the same time of day for each participant (±1 h) to minimize any influence of diurnal variation.

### Test procedures

#### Time trials

All time trials were performed in standardized laboratory conditions (temperature: 22.8 ± 1.5 C°, humidity: 40.5 ± 9.6%). Participants performed a standardized warm-up (5 min at 50% of P_max_, 3 min at 60% of P_max_ and 2 min at 70% of P_max_), after which they were given 5 min to relax and prepare for the subsequent trial. All time trials were created using the Velotron 3D Software and performed on an electromagnetically braked cycle ergometer. Throughout the trials participants were able to adjust their power output by altering their gear ratio and pedaling cadence as required. However, participants always started in the same simulated cycling gear for each test. Participants were instructed to complete distance-based trials as fast as possible, and complete the time-based trials with the highest average power output possible. No other guidance was given to them. The only feedback given during the 4 km and 20 km trials was distance covered (km and m), while the elapsed time (min:s:ms) was the only feedback given in the 4 min and 30 min trials. Both the time and distance feedback were provided as a digital display on a computer screen using the Velotron Coaching Software with all other information that is displayed on the screen blocked by paper/card. Water consumption was *ad libitum* during each time trial. Power output was averaged over every 10% of the long trials and over 25% over the short trials. All trials were supervised by the same experimenter. A recent meta-analysis recommended to use standardized verbal ancouragement in lab-based studies to ensure that participants offer maximum effort durin an endurance task (McCormick et al., 2015). As all tests were conducted by the same investigator standardized verbal encouragement was provided during the last split in each trial.

#### Physiological measures

Throughout all trials, heart rate (HR) was continuously recorded with a portable heart rate monitor and analyzed using the PolarPro Trainer 5 (Polar Electro, Kempele, Finland). Similar to power output, heart rate was averaged over 10% of the long trials and 25% over the short trials. Blood samples were drawn from the participant's forefinger at the end of each trial (1, 3, and 5 min after cessation) by pin prick and analyzed for maximum whole blood lactate concentration (Bla_peak_) using a Lactate pro2 analyser (Arkray Inc., Kyoto, Japan).

#### Psychological measures

Before each trial participants completed success motivation and intrinsic motivation scales (Matthews et al., 2001). Each scale consists of seven items (e.g., “I want to succeed on the task” and “I am concerned about not doing as well as I can”) scored on a 5-point Likert scale (0 = not at all, 1 = a little, 2 = somewhat, 3 = very much, 4 = extremely). Therefore, total scores for these motivation scales range between 0 and 28. During each trial participants were asked to report their perceived exertion (RPE) using the Borg's 6–20 scale (Borg, 1982) at 1 km and 90 s intervals during the short trials and at 2 km and 3 min intervals during the long trials. Prior to the experimental trials, participants were given standard instructions for overall RPE and were asked to report based on the degree of whole body heaviness and strain experienced during the exercise task (Borg, 1982). Partcipants were also familiarized with the RPE scale during the incremental test, during which the low and high anchor points were established using standard procedures (Borg, 1982). Since it has recently been shown that the presence of a male or female observer has a significant influence on RPE (Winchester et al., 2012) participants at both locations were asked to report their RPE by the same female investigator in each test.

### Statistical analysis

Data was normally distributed (Kolmogorov-Smirnov-Test). A paired sample *t*-test was used to test for order effects within the short and long trials as well as for differences in Bl_peak_. A one-way ANOVA was used to compare overall performance parameters between the rewarded and non-reward group in the short and long trials. Additionally, Cohen's effect sizes (*d*) and thresholds (<0.2, >0.2, 0.6, 1.2, 2.0 for trivial, small, moderate, large, and extremely large; Hopkins et al., 2009) were also used to compare the magnitude of the differences in overall performance parameters. To compare pacing, speed in all trials was additionally expressed relative to average speed (normalized mean velocity). The approach to express pacing as the difference between current and overall mean velocity is well-accepted as it better reflects the pacing pattern instead of overall performance (Abbiss and Laursen, 2008). Two-way mixed model ANOVAs (factor 1: group, factor 2: distance/time split) were used to compare power output, heart rate and RPE between groups. Change scores between splits were further calculated and analyzed via a two-way mixed model ANOVA (factor 1: group, factor 2: Δ split). Therefore, the difference in power output between splits was calculated as percentage of the preceding split (e.g., Δ split 1–2 for the difference in power output between 1 and 2 in a trial). Greenhouse-Geiser (G-G) corrections have been applied where the assumption of sphericity has been violoted. When significant main effects were observed, a Tukey *post-hoc* test was performed. *P* < 0.05 for the α error was accepted as the level of significance for statistical comparisons. Effect sizes (partial eta squared, η^2^) were calculated for the ANOVA main effects.

## Results

### Overall performance

No difference between groups was observed in P_max_ in the incremental cycling test (non-rewarded: 391.2 ± 48.2, rewarded: 397.8 ± 41.2; *p* = 0.73, *d* = 0.13). The order of the trials did not have a significant effect on overall performance in either the short (*p* > 0.74) nor the long trials (*p* > 0.50). Overall performance results for all trials are displayed in Table 2. Performance time and covered distance were not significantly different between the reward and non-reward groups during either the short (4 km *p* = 0.86, *d* = −0.07 and 6 min *p* = 0.77, *d* = −0.1, respectively) or the long trials (20 km *p* = 0.95, *d* = −0.02 and 30 min *p* = 0.80, *d* = 0.08, respectively), which was supported by trivial effect sizes (Table 2).

**Table 2:** Overall results (*n* = 23) for both short and long cycling time trials.

	**4 km**		**6 min**		**20 km**		**30 min**	
	**No reward**	**Reward**	**No reward**	**Reward**	**No reward**	**Reward**	**No reward**	**Reward**
Time (min:s)	5:40.2 ± 18.2	5:42.0 ± 18.0			29:51.1 ± 1:44.7	29:48.1 ± 1:26.7		
km			4.27 ± 0.21	4.25 ± 0.18			19.92 ± 1.38	20.05 ± 1.02
Mean difference (±95% Cl)	−1.3 ± 15.9		−0.02 ± 0.2		−2.8 ± 83.5		0.13 ± 1.1	
d	−0.07 ± 0.8		−0.1 ± 0.7		−0.02 ± 0.7		0.08 ± 0.7	
PO (W)	322.3 ± 47.6	323.8 ± 42.8	324.1 ± 46.7	320.0 ± 37.2	271.4 ± 43.9	272.9 ± 35.9	266.1 ± 39.3	267.0 ± 36.5
Mean difference (±95% Cl)	1.5 ± 39.3		−4.0 ± 36.6		1.5 ± 34.7		0.94 ± 33.0	
d	0.03 ± 0.8		−0.06 ± 0.7		0.03 ± 0.7		0.02 ± 0.8	
HR_mean_ (bpm)	178.6 ± 10.1	171.0 ± 8.7	179.7 ± 8.7	173.0 ± 7.8	175.1 ± 7.4	168.6 ± 10.9	173.2 ± 11.3	165.8 ± 16.4
RPE_mean_	17.3 ± 1.2	16.7 ± 0.9	16.9 ± 1.5	16.6 ± 0.8	16.2 ± 1.0	15.8 ± 0.8	16.0 ± 1.3	15.6 ± 0.9
Bla_peak_ (mmol/L)	15.9 ± 3.6	11.1 ± 2.5^*^	15.0 ± 3.2	10.6 ± 7.8^*^	12.1 ± 3.7	9.3 ± 2.9	11.0 ± 3.6	9.6 ± 3.2
Intrinsic motivation	25.5 ± 2.5	23.4 ± 2.5	25.0 ± 3.4	22.5 ± 3.2	24.3 ± 4.2	23.4 ± 2.7	24.6 ± 3.4	21.9 ± 2.8
d	−0.8 ± 0.8		−0.6 ± 0.8		−0.2 ± 0.7		−0.7 ± 0.7	
Task oriented motivation	23.0 ± 3.6	19.2 ± 3.6	22.1 ± 3.7	18.4 ± 3.8^*^	22.3 ± 3.8	18.4 ± 3.8	23.1 ± 2.8	19.9 ± 7.5
d	−0.9 ± 0.8		−0.2 ± 1.3		−0.9 ± 0.8		−1.1 ± 1.8	

*Mean ± standard deviation; Cohen's effect size (d)*.

* *denotes significantly different to non-rewarded group*.

### Pacing pattern

#### Pattern of power output

**Figure 1:**
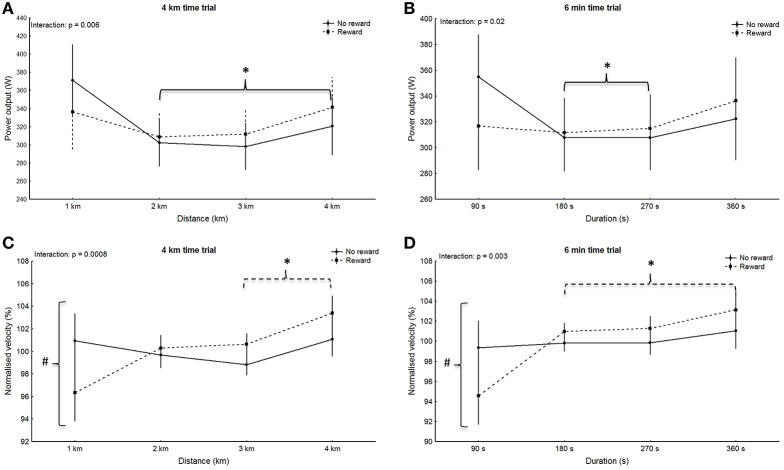
Pattern of power output **(A,B)** with corresponding normalized pacing **(C,D)** during the 4 km **(A,C)** and the 6 min **(B,D)** time trials. Means ± standard deviation. Solid line, non-rewarded; dotted line, rewarded. ^*^ indicates a significant within-trial difference to split one; ^∧^ indicates a significant within-trial difference to the last split; # indicates a significant effect between groups at this split.

**Figure 2:**
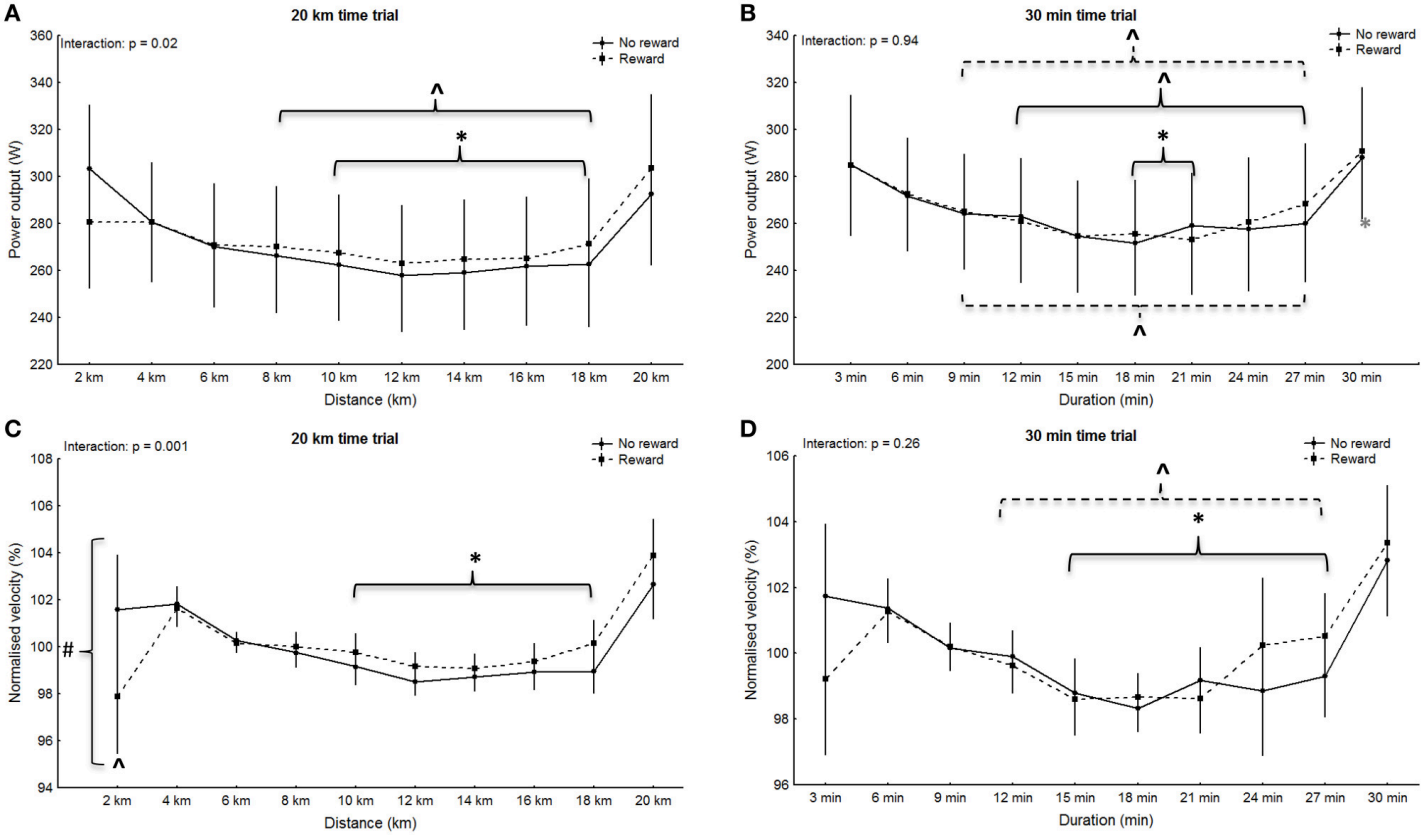
Pattern of power output **(A,B)** with corresponding normalized pacing **(C,D)** during the 20 km **(A,C)** and the 30 min **(B,D)** time trials. Means ± standard deviation. Solid line, non-rewarded; dotted line, rewarded. ^*^ indicates a significant within-trial difference to split one; ^∧^ indicates a significant within-trial difference to the last split; ^#^indicates a significant effect between groups at this split.

No significant main effect for group was observed in any of the trials (4 km: *p* = 0.94, *F* = 0.005, η^2^ = 0.0002; 6 min: *p* = 0.85, *F* = 0.04, η^2^ = 0.002; 20 km: *p* = 0.90, *F* = 0.02, η^2^ = 0.0007; 30 min: *p* = 0.95, *F* = 0.005, η^2^ = 0.0002), however main effect for split was significant for all trials (4 km: *p* < 0.001, *F* = 15.89, η^2^ = 0.43; 6 min: *p* = 0.007, *F* = 4.36, η^2^ = 0.17; 20 km: *p* < 0.001, *F* = 18.97, η^2^ = 0.47; 30 min: *p* < 0.001, *F* = 15.6, η^2^ = 0.43). Within-group *post-hoc* analysis for the non-rewarded group revealed a significantly higher power output in split one compared to all other splits during the 4 km trial (*p* < 0.001; Figure 1A). Additionally, power in split one in the 6 min trial was significantly higher compared to split two and three (*p* < 0.006; Figure 1B). In the non-rewarded group power output in the first split was significantly greater compared to split five to nine in the 20 km trial (*p* < 0.02; Figure 2A) and compared to six and seven in the 30 min trial (*p* < 0.04, Figure 2B). This group also showed a significantly higher power output in split ten compared to split four to nine in the 20 km (*p* < 0.03; Figure 2A) and the 30 min (*p* < 0.02; Figure 2B) trial. *Post-hoc* analysis in the rewarded group only revealed a significantly higher power output in the last split of the 30 min trial (split ten vs. split three to nine: *p* < 0.001; Figure 2B).

Significant main effects for split-by-group interaction were observed for the 4 km (G-G corrected: *p* = 0.006, *F* = 4.59, η^2^ = 0.18; Figure 1A), 6 min (G-G corrected: *p* = 0.02, *F* = 3.58, η^2^ = 0.15; Figure 1B) and the 20 km trial (G-G corrected: *p* = 0.02, *F* = 2.36, η^2^ = 0.10; Figure 2A), but not the 30 min trial (G-G corrected: *p* = 0.94, *F* = 0.37, η^2^ = 0.02; Figure 2B). However, *post-hoc* analysis did not confirm significant differences in single splits (*p* > 0.05). Nontheless, between group effect sizes were moderate for the first split in the 4 km (*d* = 0.44), 6 min (*d* = 0.59), and the 20 km (*d* = 0.44).

#### Pattern of normalized velocity

No significant main effect for group was observed in any of the trials (4 km: *p* = 0.82, *F* = 0.0, η^2^ = 0.0002; 6 min: *p* = 0.39, *F* = 1.0, η^2^ = 0.03; 20 km: *p* = 0.06, *F* = 4.0, η^2^ = 0.16; 30 min: *p* = 0.90, *F* = 0.0, η^2^ = 0.0007), however main effect for split was significant for all trials (4 km: *p* = 0.002, *F* = 5.65, η^2^ = 0.21; 6 min: *p* < 0.001, *F* = 9.31, η^2^ = 0.31; 20 km: *p* < 0.001, *F* = 12.84, η^2^ = 0.38; 30 min: *p* < 0.001, *F* = 8.09, η^2^ = 0.28). Within-group *post-hoc* analysis revealed a significant difference between the first split and all other splits in the 6 min trial of the rewarded group (*p* < 0.001; Figure 1D). The same split was significantly different to split three and four in the 4 km trial (*p* < 0.03; Figure 1C). *Post-hoc* analysis revealed no within-group difference between splits during the short trials of the non-rewarded group (*p* > 0.05), however the first split was significantly different to split five to nine during the 20 km trial (*p* < 0.009; Figure 2C) as well as the 30 min trial (*p* < 0.02; Figure 2D). In the rewarded group within-group differences were observed between split 10 and four to nine during the 30 min trial (*p* < 0.02; Figure 2D), whereas no difference between splits was found during the 20 km trial (*p* > 0.05).

Main effects for split-by-group interaction were observed for the 4 km (G-G corrected: *p* < 0.001, *F* = 6.30, η^2^ = 0.23; Figure 1C), 6 min (G-G corrected: *p* = 0.003, *F* = 5.01, η^2^ = 0.19; Figure 1D) and 20 km (G-G corrected: *p* = 0.001, *F* = 3.23; η^2^ = 0.13; Figure 2C), but not the 30 min trial (G-G corrected: *F* = 1.26; *p* = 0.26; η^2^ = 0.06; Figure 2D). *Post-hoc* analysis revealed a significantly slower start (split one) in the rewarded group during the 4 km (*p* = 0.02), 6 min trial (*p* = 0.006), and 20 km trial (*p* < 0.001), but no significances between groups in any other split (*p* > 0.05).

#### Split changes

Regarding changes in power output between splits no significant main effect for group was observed in the 6 min (*p* = 0.06, *F* = 3.90, η^2^ = 0.15), the 20 km (*p* = 0.08, *F* = 3.37, η^2^ = 0.14) and the 30 min trial (*p* = 0.96, *F* = 0.002, η^2^ = 0.0001), but the main effect was significant in the 4 km trial (*p* = 0.03, *F* = 5.74, η^2^ = 0.21). Main effect for split was significant for all trials (4 km: *p* = 0.001, *F* = 41.43, η^2^ = 0.65; 6 min: *p* < 0.001, *F* = 17.09, η^2^ = 0.44; 20 km: *p* < 0.001, *F* = 21.24, η^2^ = 0.50; 30 min: *p* < 0.001, *F* = 16.80, η^2^ = 0.43). Within-group *post-hoc* analysis revealed a significantly greater change in Δ split 1–2 compared to all other Δ split in the 4 km (*p* < 0.001) and the 6 min (*p* < 0.001) of the non-rewarded group (Table 3). Furthermore, the non-rewarded group showed a signifcantly larger change in power in Δ split 1–2 compared to Δ split 3–4 (*p* = 0.04), Δ split 4–5 (*p* = 0.04) as well as Δ split 6–7 to Δ split 9–10 (*p* < 0.001) in the 20 km trial (Table 3). The same Δ split was further significantly greater compared to Δ split 6–7 in the 30 min trial (*p* = 0.02, Table 3). In the rewarded group Δ split 1–2 was signifcantly smaller compared to Δ split 3–4 in the 4 km trial (*p* < 0.001, Table 3). Additionally, the increase in power output from split 9 to 10 was signifcantly greater compared to all other split changes in the 20 km trial (*p* < 0.001) and compared to Δ split 1–2 to 8–9 in the 30 min trial (*p* < 0.02, Table 3).

**Table 3:** Mean split change (Δ) of power output between splits.

**Trial**	**No reward**	**Reward**	**Effect size (d)**
**4 km**			
Δ split 1−2	−17.3 (−23.7 to 10.8)	−6.9 (−15.8 to 1.9)^*^	0.95 (±0.95)
Δ split 2−3	−1.3 (−3.0 to 0.4)^#^	1.1 (−2.1 to 4.3)	0.84 (±1.19)
Δ split 3−4	7.3 (5.2 to 9.4)^#^	9.3 (3.7 to 14.9)	0.56 (±1.66)
**6 min**			
Δ split 1−2	−12.4 (−19.8 to −4.9)	−0.5 (−9.8 to 8.8)^*^	0.94 (±0.89)
Δ split 2−3	0.2 (−3.5 to 3.9)^#^	1.3 (−2.5 to 5.0)	0.16 (±2.79)
Δ split 3−4	4.6 (1.7 to 7.6)^#^	6.4 (1.0 to 11.8)^#^	0.36 (±1.19)
**20 km**			
Δ split 1−2	−7.3 (−11.7 to −2.9)	0.9 (−6.8 to 8.7)^*^^∧^	−1.12 (±1.15)
Δ split 2−3	−3.8 (−5.3 to −2.4)	−3.7 (−5.9 to −1.5)^∧^	−0.09 (±1.03)
Δ split 3−4	−1.3 (−2.2 to −0.3)^#^	−0.1 (−2.7 to 2.6)^∧^	0.71 (±1.65)
Δ split 4−5	−1.3 (−3.3 to −0.6)^#^	−1.0 (−2.7 to 2.6)^∧^	0.09 (±0.64)
Δ split 5−6	−1.8 (−3.4 to −0.3)	−1.6 (−4.3 to 1.0)^∧^	0.06 (±1.11)
Δ split 6−7	0.4 (−1.1 to 2.0)^#^	0.7 (−2.4 to 3.9)^∧^	0.11 (±1.33)
Δ split 7−8	0.9 (−1.6 to 3.5)^#^	0.3 (−2.0 to 2.5)^∧^	−0.15 (±0.75)
Δ split 8−9	0.4 (−0.9 to 1.6)^#^	2.1 (−1.2 to 5.4)^∧^	0.80 (±1.63)
Δ split 9−10	11.6 (8.4 to 14.8)^#^	11.9 (7.0 to 16.9)	0.06 (±1.03)
**30 min**			
Δ split 1−2	−4.2 (−7.4 to −1.0)	−3.7 (−8.6 to 1.3)^∧^	0.10 (±1.03)
Δ split 2−3	−2.9 (−5.1 to −0.7)	−2.6 (−5.0 to −0.08)^∧^	0.09 (±0.63)
Δ split 3−4	−0.6 (−2.0 to 0.7)	−1.6 (−3.7 to 0.5)^∧^	−0.44 (±1.05)
Δ split 4−5	−2.8 (−4.6 to −0.9)	−2.5 (−5.1 to 0.1)^∧^	0.09 (±0.97)
Δ split 5−6	−1.3 (−2.9 to 0.4)	0.7 (−2.7 to 4.0)^∧^	0.69 (±1.28)
Δ split 6−7	2.8 (0.7 to 5.0)^#^	−0.5 (−4.4 to 3.3)^∧^	−0.92 (±1.14)
Δ split 7−8	−1.0 (−5.5 to 3.5)	3.0 (−2.4 to 8.4)^∧^	0.53 (±0.87)
Δ split 8−9	1.4 (−1.8 to 4.5)	3.2 (−1.1 to 7.6)	0.35 (±0.96)
Δ split 9−10	11.2 (8.4 to 14.0)	8.4 (4.1 to 12.8)	0.15 (±1.14)

* *denotes significantly different to non-rewarded group*.

# *denotes a significant within-trial difference to Δ split 1–2*.

∧ *denotes a significant within-trial difference to Δ split 9–10*.

Main effects for split-by-group interaction were significant for the 6 min (G-G corrected: *p* = 0.03, *F* = 3.84, η^2^ = 0.15) but no other trial (4 km: G-G corrected: *p* = 0.07, *F* = 2.90, η^2^ = 0.12; 20 km: G-G corrected: *p* = 0.07, *F* = 1.87, η^2^ = 0.07; 30 min: G-G corrected: *p* = 0.37, *F* = 1.10, η^2^ = 0.04). *Post-hoc* analysis revealed a signficantly greater change in power output from split 1 to 2 in the non-rewarded compared to the rewarded group during the 4 km (*p* = 0.02), the 6 min (*p* = 0.02), and the 20 km trial (*p* = 0.04; Table 3). *Post-hoc* analysis did not show any significant split-by-group interactions during the 30 min trial (*p* > 0.94; Table 3). Effect sizes were moderate to large for all Δ splits in the 4 km trial as well as for Δ split 1–2 and Δ split 3–4 in the 6 min trial. For the 20 km trial large effect sizes were observed for Δ split 1–2, Δ split 3–4, and Δ split 8–9, as well as for Δ split 3–4. Δ split 5–6, Δ split 6–7, and Δ split 7–8 for the 30 min trial (Table 3).

### Psychological measures

**Figure 3:**
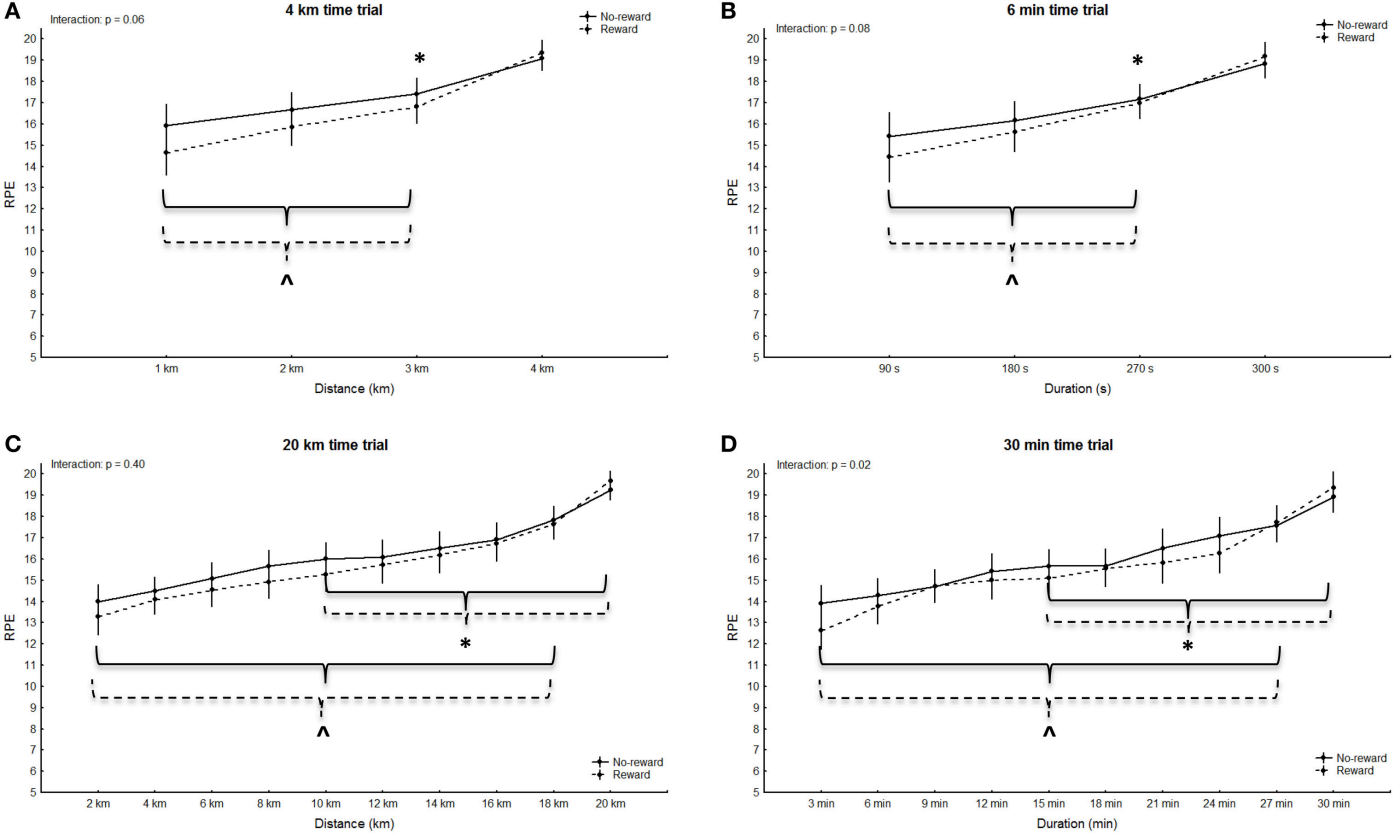
RPE during the short **(A,B)** and the long trials **(C,D)**. Means ± standard deviation. Solid line: non-rewarded; dotted line: rewarded. ^*^ indicates a significant within-trial difference to split one; ^∧^ indicates a significant within-trial difference to the last split; # indicates a significant effect between groups at this split.

No significant main effect for group was observed in any of the trials (4 km: *p* = 0.18, *F* = 1.94, η^2^ = 0.08; 6 min: *p* = 0.53, *F* = 0.41, η^2^ = 0.02; 20 km: *p* = 0.34, *F* = 0.95, η^2^ = 0.04; 30 min: *p* = 0.43, *F* = 0.64, η^2^ = 0.03), however the main effect for split was significant in all trials (4 km: *p* < 0.001, *F* = 68.38, η^2^ = 0.77; 6 min: *p* < 0.001, *F* = 93.71, η^2^ = 0.82; 20 km: *p* < 0.001, *F* = 96.58, η^2^ = 0.82; 30 min: *p* < 0.001, *F* = 107.98, η^2^ = 0.84). RPE in both groups was significantly higher at the last split compared to all other splits in all trials (4 km: *p* < 0.02; 6 min: *p* < 0.001; 20 km: *p* < 0.005; 30 min: *p* < 0.006). Additionally, in the short trials of both groups RPE was significantly higher in split three compared to split one (4 km: *p* < 0.001; 6 min: *p* < 0.008; Figures 3A,B). RPE in the second half of the long trials was further significantly higher compared to the first half in both groups (20 km: *p* < 0.004; 30 min *p* < 0.004; Figures 3C,D). No significant main effects for split-by-group interaction in RPE was observed in the 4 km (G-G corrected: *p* = 0.06, *F* = 2.58, η^2^ = 0.11), 6 min (G-G corrected: *p* = 0.08, *F* = 2.35, η^2^ = 0.10) and 20 km trials (G-G corrected: *p* = 0.40, *F* = 1.05, η^2^ = 0.05) (Figures 3A–C). A significant main effect for split-by-group interaction was observed in the 30 min trial (G-G corrected: *F* = 2.35; *p* = 0.02; η^2^ = 0.10), however, *post-hoc* analysis did not confirm significant differences in single splits (*p* > 0.75; Figure 3D). The rewarded group showed a significantly lower task-oriented motivation prior to the 20 km trial (*p* = 0.02, Table 2). Even though no significant differences in motivation were observed prior to the remaining trials (*p* > 0.05) moderate to large effect sizes indicate reduced intrinsic and task-oriented motivation in the rewarded group (Table 2).

### Physiological measures

No significant main effect for group was observed in heart rate in any trial (4 km: *p* = 0.07, *F* = 3.65, η^2^ = 0.15; 6 min: *p* = 0.07, *F* = 3.67, η^2^ = 0.15; 20 km: *p* = 0.11, *F* = 2.84, η^2^ = 0.12; 30 min: *p* = 0.22, *F* = 1.63, η^2^ = 0.07), however main effect for split was significant in all trials (4 km: *p* < 0.001, *F* = 37.24, η^2^ = 0.64; 6 min: *p* < 0.001, *F* = 48.70, η^2^ = 0.70; 20 km: *p* < 0.001, *F* = 32.45, η^2^ = 0.61; 30 min: *p* < 0.001, *F* = 23.16, η^2^ = 0.52). As such, heart rate in both groups was signficantly higher in the last split compared to all other splits (*p* < 0.002). No significant main effects for split-by-group interaction in heart rate were observed in the 4 km (G-G corrected: *p* = 0.05, *F* = 2.71, η^2^ = 0.11), 6 min (G-G corrected: *p* = 0.14, *F* = 1.88, η^2^ = 0.08) and 30 min trial (G-G corrected: *p* = 0.38, *F* = 1.08, η^2^ = 0.05) A significant main effect for split-by-group interaction was observed in the 20 km trial (G-G corrected: *p* = 0.04, *F* = 2.01 η^2^ = 0.09), however, *post-hoc* analysis did not confirm significant differences in single splits (*p* > 0.58). Bla_peak_ concentration was significantly lower after both short trials in the rewarded group compared to the non-rewarded group (*p* < 0.04, Table 2).

## Discussion

The novel finding of the present study was that despite similar performance between groups, monetary rewards seem to alter pacing upon commencement of both short and long cycling time trials. Participants in the non-rewarded group adopted a parabolic shaped pattern, whereas the rewarded group started all trials more conservatively. The current results are in contrast to findings by Hulleman et al. (2007) who observed no changes in pacing when participants were spontaneously rewarded with $100 if they out-performed their best 1,500 m cycling performance. However, those rewards were not declared until just before the trial. The greater period of time before the trial and the larger reward offered in the present study may have been responsible for the different outcomes. Several current theories suggest that the athlete's pacing pattern is dependent on a pre-established template that determines power output pattern during an event, and developed through extensive training and competition experience (Foster et al., 2009; Renfree et al., 2014; Skorski and Abbiss, 2017). This is not readily reconciled with the current findings, wherein monetary rewards appear to have altered pacing. Typically, under laboratory conditions, studies report a parabolic-shaped pacing in cycling time trials (Stone et al., 2011; Thomas et al., 2011). The rewarded group, however, started conservatively resulting in a “slow-fast” pacing pattern in three out of four trials. Indeed, whilst cyclists in the non-rewarded group started their trials at or above their average speed (100.0–101.7%) rewarded participants commenced each trial below their average speed (94.6–99.2%).

It has been proposed that pacing might be seen as a constant decision making process in which an athlete decides to reduce, increase or maintain efferent neural control depending on the perceived benefits to be obtained from each alternative (Renfree et al., 2014). When the perceived reward is potentially large, an athlete would be prepared to risk a greater likelihood of suffering severe performance decrements or even failure of physiological systems (e.g., a collapse before the finish line) (Renfree et al., 2014). Within this hypothesis, investing effort into an action will only be considered worthwhile if the expected outcome provides benefits that outweigh the expected energetic costs (Renfree et al., 2014). Since overall performance was almost identical in both groups, the monetary reward given might not have been perceived as important enough to risk high effort expenditure. Another explanation may be that as, money, status, recognition and lifestyle are so closely associated with professional sports and these external rewards might become “internalized” by highly trained athletes (Keegan et al., 2014). As such, external inducements might be viewed as normal in these environments and athletes therefore do not experience the same negative reward perceptions (e.g., feeling of losing control, being manipulated) (Chantal et al., 1996; Mallett and Hanrahan, 2004). However, as participants in the current study cannot be classified as “elite” this explanation might be rather speculative. Future research comparing elite, professional and amateur athletes is needed in order to better understand the role of reward on performance in these groups.

An alternative explanation would be that pacing is sufficiently cognitively demanding to be influenced by an extrinsic motivator; an effect which has been demonstrated in psychological research where only extremely simple tasks appear to benefit from external incentives (Bijleveld et al., 2011a; Zedelius et al., 2011). As such, pacing might involve a constant monitoring-and-reacting process, as opposed to simply executing a pre-determined template without conscious regulation. Thus, this monitoring process would be open to disruption by the introduction of incentives. Our findings in this respect are suggestive of differences in intrinsic and/or task oriented motivation, but the large variability suggests a more sensitive measurement approach would be needed to more adequately capture the role of motivation in the observed effect on pacing. It might also be speculated that it is more difficult to monitor, detect and interpret bodily signals of exertion at the beginning of the task. Indeed, physiologically there is a delay between muscle force production, metabolic response and cognitive recognition within the brain (St. Clair Gibson et al., 2006). Thus, cyclists might have adjusted their pace once afferent feedback was perceived to be different to achieve their “normal” performance. While motivational intensity theory alone may have predicted that a stronger incentive would increase performance (Brehm and Self, 1989), it may be more difficult to reconcile with the results observed.

The above interpretation is further consistent with recent psychological research where the differences in the starting strategy may reflect the “ironic effect” of an increased concentration on the actual task in the rewarded group due to the awareness of the prize money (Zedelius et al., 2011). As such, it is possible that rewards might change the way incoming information is processed and hence how people deal with a task when rewards reach consciousness (Bijleveld et al., 2010, 2011a). People might reflect more on “what is at stake” and thus focus their concentration on the specific task that is instrumental in attaining the perceived reward (Bijleveld et al., 2011a; Zedelius et al., 2014). This “enhanced concentration on task information” might interfere with task performance, when irrelevant information is also prioritized from incoming information (Bijleveld et al., 2011a). The introduction of external motivators may change the way that motivation is regulated at more conscious, cognitive levels, and thus altering the processing/interpretation of affective and physiological signals. As such, cyclists might have concentrated more on performing well at the start of each trial which conversely resulted in a slower starting pace as physiological signal feedback might have been less present. Indeed, it has been recently proposed that even though distractive strategies tend to reduce effort perceptions this can also result in a slower-than-optimum pace (Brick et al., 2016). In this regard, Brick et al. (2016) proposed a metacognitive framework of attentional focus and cognitive control for endurance performance regulation. These metacognitive skills are planning prior to performance (cognitive strategies), monitoring during performance (thinking and task completion), and reviewing and evaluation after performance (Brick et al., 2016). Indeed, metacognitive planning may involve proactive goal setting and establishing a pacing pattern (Brick et al., 2015). As athletes seem to prioritize sensory information to optimize performance (Brick et al., 2015) it might be speculated that cyclists' cognitive planning focused on the monetary reward at the start of the trial, however monitoring of internal sensory sources of information later changed metacognitive feelings during the trial. Indeed, while monitoring and control can occur at an implicit, subconscious level, conscious control is engaged when metacognitive feelings form an awareness of the task (e.g., pace is too easy) that requires an appropriate response (Brick et al., 2016) (e.g., increase of power output after the first split).

Additionally, growing body of psychological literature shows that increased task focus can hurt performance, and support the specific idea that such negative performance effects are rooted in consciousness (Bijleveld et al., 2011a). Moreover, rewarding performance with money might induce people to become more concerned with doing well and more self-conscious about an activity which should be automatic (Camerer and Hogarth, 1999). When pressure to perform is high, people often perform worse than without this pressure (“choking under pressure”) probably because conscious reflection about the reward distracts their attention from the task (Beilock and Carr, 2001). Thus, the degree of central motor drive at the start, determined from the memory of prior experience, might have had less influence due to the presence of the extrinsic motivator. To summarize, it might be speculated that cyclists concentrated more on the actual task at the start of each trial as they focused on the money at stake. However, after internal sensory feedback changed metacognitive feelings during the trials cyclists adjusted their pace to their “normal” pattern to achieve optimal performance.

## Limitations

The present study was conducted as a parallel-group design with a randomized order of the trials. Different participants were selected for the reward and non-reward groups, thus it has to be considered that the significant interaction effect in all the trials might be due to the independence of the sample. Indeed, training amount was slightly lower in the rewarded compared to the non-rewarded group (15,600 ± 7062 km/y vs. 13,100 ± 5,253 km/y). Prior experience is an important factor in regulating endurance performance, thus the slightly lower training experience of the rewarded group might have influenced the outcomes of the present study (Wilson et al., 2012). Unfortunately, it was not possible to conduct this research in a randomized cross-over design given that it would not have been possible to blind participants to the study purpose. Additionally, since participants were from different locations it is possible that the specifics of training or competition may differ in each location thereby influencing the outcomes of the present study. However, the unusual negative pacing displayed by the rewarded group was different to the non-reward group in three out of four trials and thus it seems likely that such differences are due to the monetary reward. Furthermore, overall performance did not differ between groups in any of the trials demonstrating that groups were well matched. It is further noteworthy that the motivational questionnaire used in the current study might arguably not be the best version to assess different motivational states. Therefore, future research should aim at evaluating the influence of (monetary) incentives on different aspects of motivation, such as intrinsic and extrinsic motivation.

Furthermore, a familiarization trial was not included in the study. This is a limitation since variability of time trial performance is lower between a second and a third trial compared to the first and the second one (Stone et al., 2011; Thomas et al., 2011) However, to do this a familiarization session for every trial would have been necessary. This would not have been feasible due to logistical constraints. Additionally, the randomized and counterbalanced order of the trial should reduce the likelihood of an order effect.

As trials were separated by 3–6 days it could be speculated that the female participants menstrual cycle phase influenced the results. Unfortunately, information regarding the menstrual cycle was not recorded throughout the study. However, since performance was not different between groups it is likely that any influences of menstrual cycle on the outcomes of this study were minimal. Additionally, between-group effects were analyzed instead of within-participant differences in performance. Therefore, within this study it would have been necessary to match the female participants according to their cycle phase, which was not possible due to organizational restraints. Regardless, to the author's knowledge there is currently no research indicating that the menstrual cycle influences pacing pattern of female cyclists. We therefore hypothesis that any influence of menstruation on the overall outcomes of this study were minimal.

In conclusion, the new finding of this study was that an external reward seems to influence pacing at commencement of short and long cycling time trials. Non-rewarded participants adopted a parabolic shaped pattern, whereas the rewarded group started all trials more conservatively. Thus, presenting an external motivator might have a negative influence on motivation, which is to be an important factor for athletic success. It remains unclear if the rather “unusual” pacing pattern detected in this study actually resulted in rewarded participants demonstrating an improved, similar or reduced performance compared to a best ever performance. Future research should focus on the influence of various external motivators, (e.g., competitors, coaching behavior) to get a better insight into the relationship between motivation and performance.

## Ethics statement

Human Research Ethics Committee of the Edith-Cowan University in Perth, Australia—approval Number: “11209” Full address: Human Research Edith-Cowan University Ethics Committee, 270 Joondalup Drive, Joondalup WA 6027, Australia.

## Author contributions

SS developed the study together with CA and its design, she supervised the study, interpreted the results and provided the first draft of the manuscript. KT supervised the study, provided technical equipment in Canberra and contributed to the data processing as well as the discussion CA participated in the study design, recruited the participants in Perth, and contributed to drafting the manuscript. RK and TM contributed to the discussion of the findings. All authors critically revised the manuscript for important intellectual content and gave final approval.

### Conflict of interest statement

The authors declare that the research was conducted in the absence of any commercial or financial relationships that could be construed as a potential conflict of interest.
